# aiGeneR 3.0: an enhanced deep network model for resistant strain identification and multi-drug resistance prediction in *Escherichia coli* causing urinary tract infection using next-generation sequencing data

**DOI:** 10.3389/fgene.2025.1651917

**Published:** 2025-10-23

**Authors:** Debasish Swapnesh Kumar Nayak, Abhilash Pati, Amrutanshu Panigrahi, Mudassir Khan, Bayan Alabdullah, Santanu Kumar Sahoo, Bibhuprasad Sahu, Abrar Almjally, Saurav Mallik, Tripti Swarnkar

**Affiliations:** ^1^ Department of Computer Science & Engineering, Siksha “O” Anusandhan (Deemed to be University), Bhubaneswar, Odisha, India; ^2^ Department of Computer Science and Engineering, Centurion University of Technology and Management, Bhubaneswar, Odisha, India; ^3^ Department of Computer Science and Engineering, Indian Institute of Technology, Bhilai, Chhattisgarh, India; ^4^ Department of Computer Science, College of Computer Science, Applied College Tanumah, King Khalid University, Abha, Saudi Arabia; ^5^ Jadara University Research Center, Jadara University, Irbid, Jordan; ^6^ Department of Information Systems, College of Computer and Information Sciences, Princess Nourah Bint Abdulrahman University, Riyadh, Saudi Arabia; ^7^ Department of Electronics and Communication Engineering, Siksha “O” Anusandhan (Deemed to be University), Bhubaneswar, Odisha, India; ^8^ Symbiosis Institute of Technology, Hyderabad Campus, Symbiosis International University, Pune, India; ^9^ College of Computer and Information Sciences, Imam Mohammad Ibn Saud Islamic University (IMSIU), Riyadh, Saudi Arabia; ^10^ Department of Environmental Health, Harvard T H Chan School of Public Health, Boston, MA, United States; ^11^ Department of Pharmacology & Toxicology, University of Arizona, Tucson, AZ, United States; ^12^ Department of Computer Application, National Institute of Technology, Raipur, India

**Keywords:** deep learning, machine learning, next-generation sequencing, antimicrobial resistance, antibiotic resistance genes

## Abstract

**Background:**

Infectious diseases pose a global health threat, with antimicrobial resistance (AMR) exacerbating the issue. Considering *Escherichia coli* (*E. coli*) is frequently linked to urinary tract infections, researching antibiotic resistance genes in this context is essential for identifying and combating the growing problem of drug resistance.

**Objective:**

Machine learning (ML), particularly deep learning (DL), has proven effective in rapidly detecting strains for infection prevention and reducing mortality rates. We proposed aiGeneR 3.0, a simplified and effective DL model employing a long-short-term memory mechanism for identifying multi-drug resistant and resistant strains in *E. coli*. The aiGeneR 3.0 paradigm for identifying and classifying antibiotic resistance is a tandem link of quality control incorporated with DL models. Cross-validation was adopted to measure the ROC-AUC, F1-score, accuracy, precision, sensitivity, specificity, and overall classification performance of aiGeneR 3.0. We hypothesized that the aiGeneR 3.0 would be more effective than other baseline DL models for antibiotic resistance detection with an effective computational cost. We assess how well our model can be memorized and generalized.

**Results:**

Our aiGeneR 3.0 can handle imbalances and small datasets, offering higher classification accuracy (93%) with a simple model architecture. The multi-drug resistance prediction ability of aiGeneR 3.0 has a prediction accuracy of 98%. aiGeneR 3.0 uses deep networks (LSTM) with next-generation sequencing (NGS) data, making it suitable for novel antibiotics and growing resistance identification in the future.

**Conclusion:**

This work uniquely integrates SNP-level insights with DL, offering potential clinical utility in guiding antibiotic stewardship. It also enables a robust, generalized, and memorized model for future use in AMR analysis.

## 1 Introduction

One of the biggest concerns for global public healthcare is the issue of diseases brought on by bacteria that are resistant to antibiotics, often known as antimicrobial resistance (AMR). According to estimates from the World Health Organization (WHO), there were over 700,000 fatalities from drug-resistant illnesses in 2019, and that number might increase to 10 million deaths by 2050 (Sharma et al., 2024; Chandra et al., 2021). Identifying antibiotic resistance genes (ARGs) is important for discovering the patterns of AMR and plays a key role in personalized treatment and drug discovery.

**FIGURE 1:**
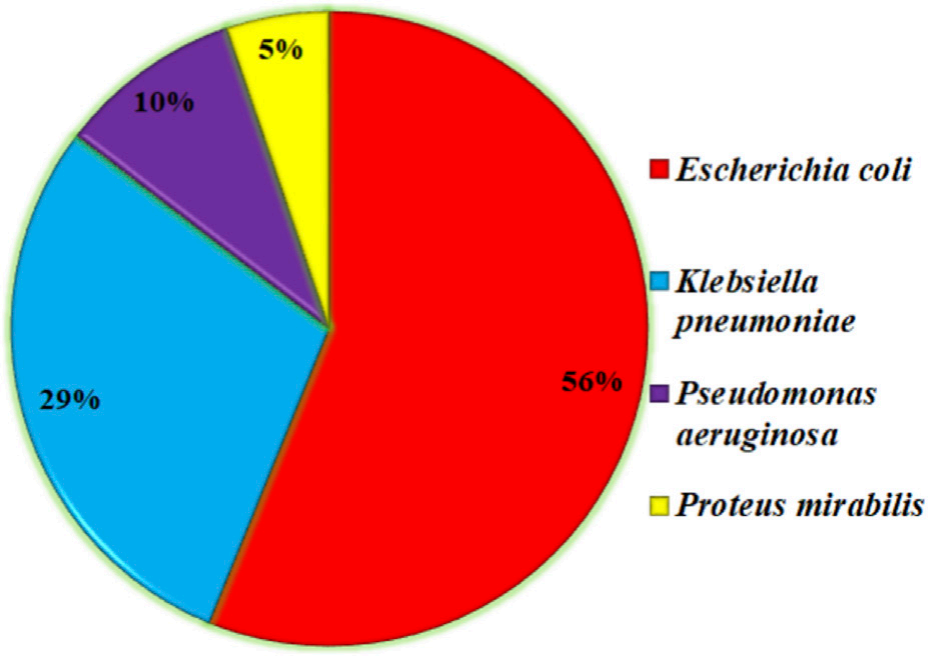
Most UTI cases are with selected bacteria.

Urinary tract infections (UTIs) are among the many infectious diseases that pose a serious threat to world health (Tan and Chlebicki, 2016). *Escherichia coli* (*E. coli*) bacteria are the main cause of UTIs, which affect millions of people each year (Vasudevan, 2014). If left untreated, many infections that affect the urinary system carry the potential to cause consequences like kidney damage. The problem is heightened by the advent of antibiotic resistance in *E. Coli* strains (Totsika et al., 2012), which restricts available treatments and calls into question accepted ideas of antimicrobial stewardship (Niranjan and Malini, 2014). *E. coli* is the primary cause of UTIs and provides a statistical analysis of other bacteria that can cause UTIs, as shown in a short research conducted in the northern region of India. *E. coli* (76.60%) was the most common gram-negative bacterium among the 47 positive isolates out of a total of 83 positive samples (Das et al., 2018). As shown in Figure 1, *E. coli* is the main cause of UTI in more than 53% of the cases, which is significant and needs to be addressed in the AMR pattern, antibiotic-resistant strains, and ARGs in *E. coli* for further effective drug development.

The robust identification of antibiotic resistance determinants and their curation in specialized databases has been made possible by the growing availability and affordability of whole-genome sequencing data from clinical strains. Computational techniques can then search these resources for known causative genes, given the sequence from a new strain (McArthur et al., 2013; Zankari et al., 2012; Stoesser et al., 2013). By detecting mutations, examining entire genomes, and pinpointing particular resistance genes, the genetic study of *E. coli* shows antibiotic resistance patterns. To effectively tackle the global challenge of antibiotic resistance, this technique aids in understanding the genetic basis of resistance, tracking its spread, and predicting emerging patterns. This information guides focused treatments for antibiotic stewardship (Truswell et al., 2023; Wilson et al., 2016; Malekian et al., 2022). Antibiotic resistance, particularly in bacteria like *E. coli*, makes urinary tract infections (UTIs) a serious concern to human health. UTIs are among the most prevalent bacterial illnesses worldwide, with millions of cases occurring yearly. UTIs can cause serious side effects such as kidney infections, sepsis, and long-term harm to the urinary system if they are not addressed. The danger is exacerbated by introducing strains resistant to antibiotics, particularly in *E. coli* (Reza Asadi et al., 2019; Jafri et al., 2014; Bryce et al., 2016).

**FIGURE 2:**
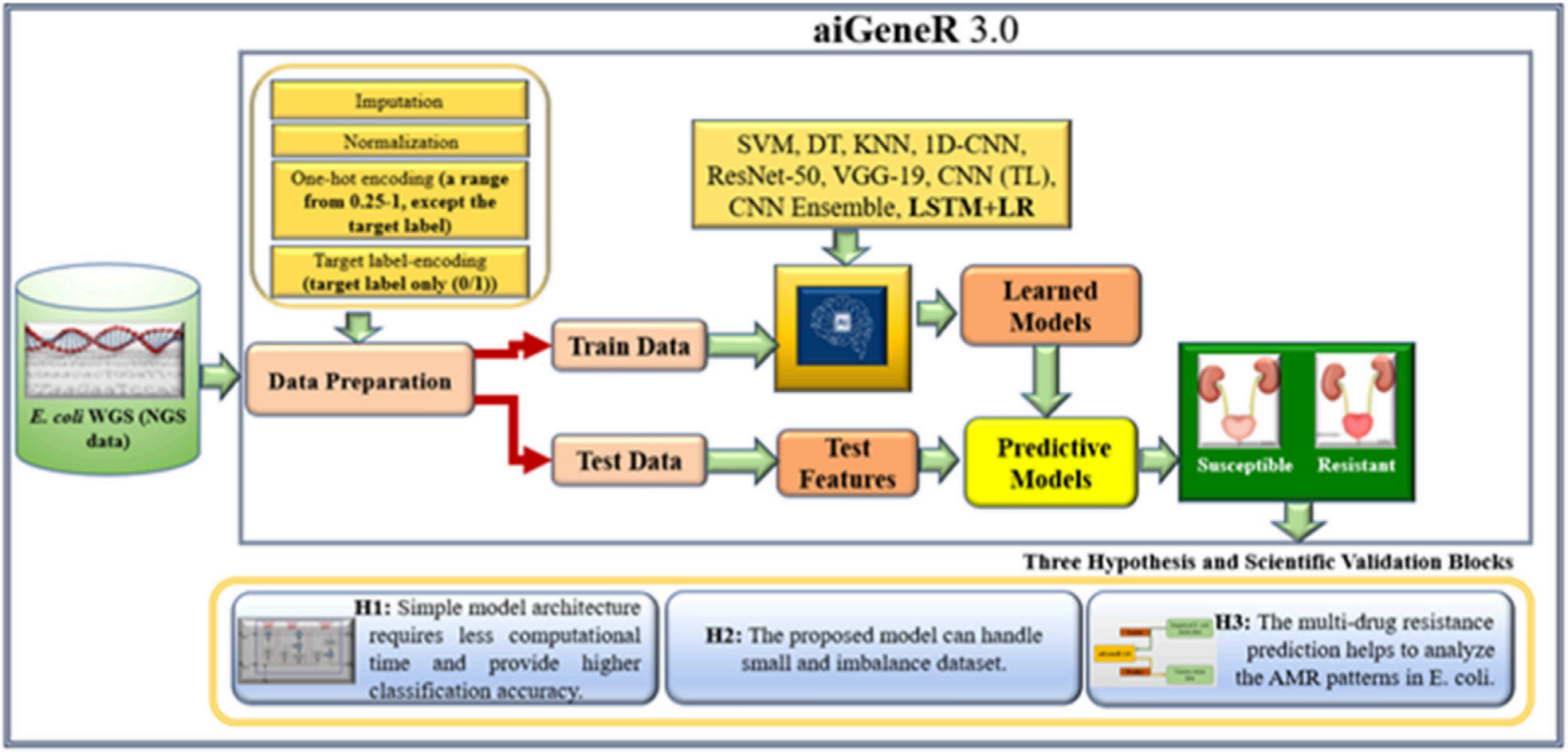
The overall architecture of our study.

Novel approaches are needed to address the growing epidemic of antibiotic resistance in urinary tract infections (UTIs). Through genetic insights, DNA data advances several sectors, including disease diagnosis, customized medicine, AMR analysis, and microbial diversity study (Satam et al., 2023). Due to its capacity to handle high-dimensional data, identify intricate correlations, and integrate various data sources, deep learning (DL) performs very well when evaluating DNA sequencing data for the identification of antibiotic resistance (Lueftinger et al., 2021; Shi et al., 2019). DL is a revolutionary method that reduces the need for wasteful antibiotic treatment by providing precision medicine through the identification of distinct resistance profiles (Bryce et al., 2016; Taylor et al., 2018). Real-time decision assistance is empowered by DL, allowing for quick and knowledgeable antibiotic selection decisions. Additionally, it makes it easier to identify new resistance trends early on, which supports preventative measures (Ren et al., 2022a; Ren et al., 2022b). This work holds the potential to completely transform the way that UTIs are managed and the identification of resistance patterns in *E. coli* utilizing the next-generation WGS data, providing efficient solutions to the ever-changing problem of antibiotic resistance. We proposed our aiGeneR 3.0 model, which can identify the multidrug resistance genes in *E. coli*. In our work, we deal with a highly imbalanced and small dataset to assess the efficacy of our aiGeneR 3.0 model. We also compare the performance of aiGeneR 3.0 with well-accepted state-of-the-art ML and DL models. The generalization of our model boosts the adaptability and robustness. The simplified architecture and less computational time are the major advantages of our aiGeneR 3.0 model. We hypothesized that the aiGeneR 3.0 can reduce the cost and time for multi-drug resistance identification utilizing the WGS data. The dataset (NGS single-nucleotide polymorphism (SNP) WGS) utilized in this work is small and imbalanced; still, our aiGeneR 3.0 performs exceptionally well; the ROC value achieved during the deployment phase has already proven this. The detailed architecture of our study is shown in Figure 2.

The following describes the paper’s structure and major contributions. The relevant work for classifying and identifying *E. coli* antibiotic resistance is included in Section 2 to set up our research pipeline. We go over the aiGeneR 3.0 content and overall design in Section 3. The AI models and the experimental technique are presented in Section 4. Section 5 has the experimental results presentation. The validation and discussion of our aiGeneR 3.0 outcome are conducted in Section 6 and Section 7, respectively. We benchmarked our aiGeneR 3.0 in Section 8, and Section 9 held the conclusion.

## 2 Literature surveys

Researchers Moradigaravand et al. (2018) used gradient-boosted decision trees to achieve a 91% success rate in predicting antibiotic resistance in 1,681 *Escherichia coli* strains. Researchers found that using population structure and gene content greatly improved prediction accuracy. Based on these findings, machine learning (ML) shows promise as a clinical tool for identifying antibiotic resistance. Introduced by Arango-Argoty et al. (2018), the DeepARG-SS model outperformed conventional approaches with a recall of 91% and an accuracy of 97% over 30 antibiotic categories. Applying the DeepARG-LS model to the MEGARes database confirmed its great recall and accuracy. When used in conjunction with the DeepARG-DB database, these models allow for more precise gene identification by producing predictions of antibiotic resistance genes. The difficulties and limits of using ML to forecast antibiotic resistance were addressed by Boolchandani et al. (2019). In order to improve the accuracy of predictions, the study highlighted the necessity for extensive databases that connect resistance genes to test results. The significance of continuously improving computational methods to combat antibiotic resistance was highlighted by recognizing Resfams, Resfinder, and CARD as effective techniques for finding resistance genes. Among the multi-label classification models used by Ren et al. (2022a) to forecast *E. coli* multi-drug resistance, the ECC model proved to be the most accurate. In order to have a whole picture of resistance, the study stressed the significance of non-chromosomal genetic variables. Researchers Gunasekaran et al. (2021) used DL methods to classify DNA sequences, successfully determining the origins of viruses and DNA mutations with a high degree of accuracy. This research proved that DL could be useful for a variety of genetic analysis, drug discovery, and viral identification tasks. The accuracy of antimicrobial resistance predictions for underrepresented groups was greatly enhanced by the deep transfer learning model put forth by Ren et al. (2022b) while dealing with tiny, imbalanced datasets. Rapid diagnosis and focused therapies could both benefit from this strategy.

Over the last decade, various tools, quality control pipelines, and AI models have been gaining attention in AMR analysis. The AMR mechanism is too complex and requires trained manpower to access the laboratory test for the identification of resistance patterns, resistance strains, and multi-drug resistant percentages. In addition to this, the procedure of resistant strain identification is associated with massive cost and time. However, AI models have been found to perform well compared to traditional approaches for resistant strain identification. It was also found that existing AI studies for resistant strain identification lack a comparative analysis that includes ensemble and simplified model architectures. In addition to this, the computational cost associated with the resistant strain identification is still an open issue. This study aims to bridge this gap and provide evidence for the superiority of ensemble-based DL, transfer learning, and solo simplified architecture-based DL models regarding prediction accuracy and computational cost. Additionally, it is observed from the literature that researchers used transfer learning (TL) on a small dataset to identify the resistant strains. We aim to achieve a more effective outcome with less model complexity and computational time. Interventional studies involving animals or humans, as well as other studies that require ethical approval, must list the authority that provided approval and the corresponding ethical approval code.

In this section, where applicable, authors are required to disclose details of how generative artificial intelligence (GenAI) has been used in this paper (e.g., to generate text, data, or graphics, or to assist in study design, data collection, analysis, or interpretation). The use of GenAI for superficial text editing (e.g., grammar, spelling, punctuation, and formatting) does not need to be declared.

## 3 Materials and methods

The methods, resources, and materials used in this study to accomplish the study’s goals are described in this section. This section seeks to present a clear and thorough explanation of the experimental design, data collection, and data analysis methods.

### 3.1 Dataset

The *E. coli* WGS dataset utilized in this study is openly available and was collected from GitHub (2025), and Moradigaravand et al. (2018). Both these datasets have the susceptible and resistant information of the WGS of the *E. coli* K-12 strain. Due to the common association between these mutations and increased antibiotic resistance in both environmental and clinical contexts, the double-mutated *E. coli* genome dataset was chosen for its practical and clinical importance. The genetic variety of resistant strains is reflected in this dataset, which captures changes linked to resistance to several classes of antibiotics. This provides valuable insights into the complicated processes of resistance. Previous studies have mostly concentrated on single mutations or resistance genes specific to individual isolates; this work fills a significant void by shifting the focus to double mutations.

### 3.2 Dataset collection

We employed two datasets of *E. coli* in this study, which included WGS, SNP, and resistance-susceptible data for four antibiotics: gentamicin (GEN), cefotaxime (CTX), ciprofloxacin (CIP), and ceftazidime (CTZ), as shown in Table 1. The first dataset has 809 *E. Coli* strains, which are generated by Ren et al. (2022b). Clinical samples from both humans and animals were used to get the isolates. Using the VITEK^®^ 2 system (bioMérieux, Nurtingen, Germany), antimicrobial susceptibility testing was carried out, and results were evaluated by EUCAST criteria. The proportion of isolates resistant to CTX, GEN, CTZ, and CIP is 23%, 44%, 34%, and 45% in that sequence.

**TABLE 1:** Strain distribution to all the studied antibiotics.

Antibiotics	GEN	CTZ	CTX	CIP
# Susceptible	188	276	358	366
# Resistance	621	533	451	443
Total	809	809	809	809

**FIGURE 3:**
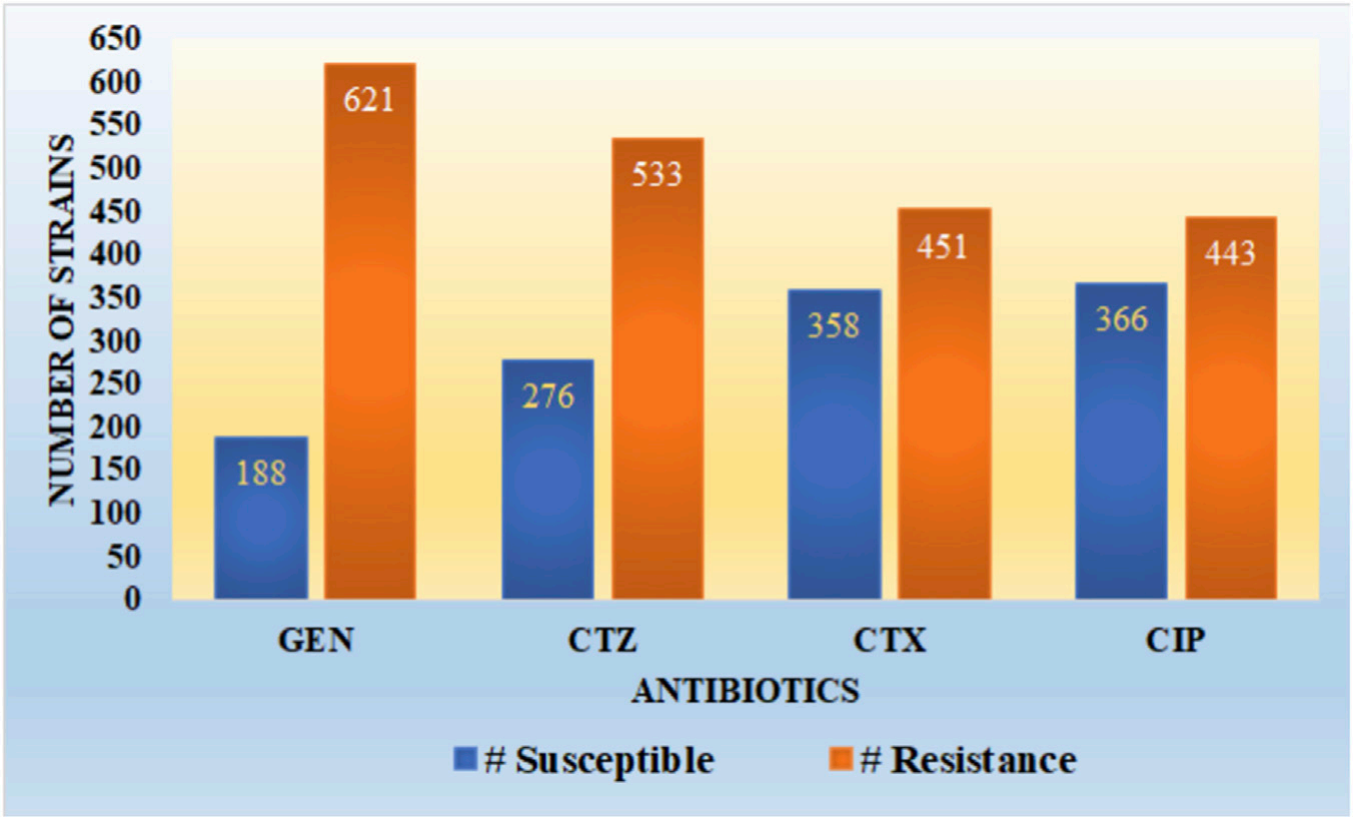
Susceptible and resistant strains for all four antibiotics.

It is observed from Figure 3 that the dataset utilized in our work has a high imbalance ratio of resistance-susceptible strains for GEN and CTZ antibiotics, with a slight improvement in the case of inconsistency for CTX and CIP antibiotics. There is a significant imbalance ratio in susceptible (S): resistant (R) of 1:3 and 1:2 in the case of GEN and CTZ antibiotics, respectively. While considering all four antibiotics, the ratio of S: R is 1:2 (1,188:2048).

### 3.3 Quality control

Data quality is the key to various AI model performances (Nayak et al., 2022; Nayak et al., 2023; Swain et al., 2023). Ren, Y. et al. developed the dataset (GitHub, 2025) to preprocess the raw WGS data; it uses BWA-MEM, and clean reads were mapped to the *E. coli* reference genome (*E. coli* K-12 strain, MG1655) after low-quality reads were filtered using fastp (v0.23.2) (Chen et al., 2018). By extracting reference and variant alleles and combining isolates according to reference allele positions, single-nucleotide polymorphisms (SNPs) were found using bcftools (v1.14) (Danecek et al., 2011; Li and Durbin, 2009). Preserving alleles that were found to be variations in more than half of the samples and creating an SNP matrix. One-hot encoding transformed the matrix into a binary format for further ML analysis.

### 3.4 Data preparation

This phase is the most crucial and contributes the most toward the model’s performance (Nayak et al., 2024; Mohanty et al., 2023). We utilized the dataset developed by GitHub (2025) for our study. Hence, we restructured the dataset to meet our study objective. The one-hot encoding in the original data ranges from 1-4, while in our study, we modified this to 0.25-1. In addition, we aim to study the effect of this one-hot encoding on computational costs.

### 3.5 Proposed model aiGeneR 3.0

**FIGURE 4:**
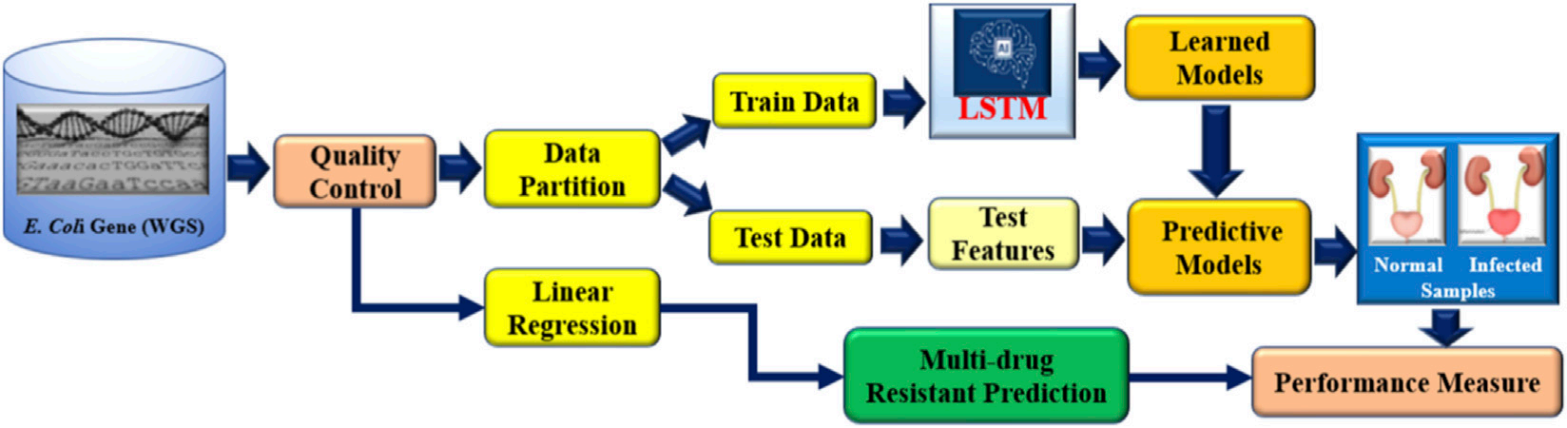
Overall architecture of our proposed aiGeneR 3.0 model.

To identify *E. coli* strains that have gained resistance, we created the state-of-the-art aiGeneR 3.0 model; this model is based on DL and ML. The approach we have created is multi-staged and uses modern techniques to boost accuracy and robustness. Beginning with processed Next-Generation Sequencing (NGS) WGS data (GitHub, 2025), which offers a solid foundation for comprehensive inquiry, we use it as our primary source. To prepare the dataset, we use the quality control (QC) pipeline that we developed before (Nayak et al., 2022). In the final phase, highly-trained deep neural networks (LSTM) and Linear regression (LR) are used to reliably identify susceptible and resistant bacteria and to forecast the likelihood of multi-drug resistance in any given strain that shows resistance to any of the four antibiotics under investigation. The design and execution of aiGeneR 3.0 are depicted in Figure 4. To describe the association between gene regulation variables and resistance extent, we used Linear Regression with least-squares optimization to reduce prediction errors and identify resistance-associated markers. Linear Regression provided a baseline prediction framework for deep learning model comparison, making the aiGeneR 3.0 pipeline robust in multidrug resistance categorization. Ultimately, a comprehensive evaluation of its efficacy using a predetermined set of assessment measures ensures the model’s reliability. Biological confirmation also adds credence to its real-world utility. The aiGeneR 3.0 model is an all-inclusive and potent tool that could change the game when finding *E. coli* antibiotic resistance.

### 3.6 Algorithm: the proposed model aiGeneR 3.0

Step 1 Read the data
    Gather *E. coli* whole-genome sequencing data using NGS.
    Use fastp for sequencing reads and quality assurance (Chen et al., 2018).
    Align the filtered reads using BWA-mem.
    Adopt Bfctools for calling variants (Danecek et al., 2011).
    Sort and filter the aligned reads using Samtools (Li and Durbin, 2009)
    Let ED be the processed dataset containing SNPs. $ED=\left\{{ed}_{1},{ed}_{2},{ed}_{3},\ldots \ldots ,{ed}_{n}\right\}$

Step 2 Preparing the data
    Align up (A) the data and eliminate duplicates.
    
A=allign ED

    To remove duplicates, $\hat{A}=remove\;dulpicates\;\left(A\right)$

Step 3 Data Engineering
    Use a one-hot encoding method (OH_E_) as ${OH}_{E}=OneHotEncoade\;\left(\hat{A}\right)$ .
    Decide on normalized values between 0.25 and 1 as follows: Equation, $=0.25+0.75\;X\;\frac{{OH}_{E}-\mathrm{Min}\left({OH}_{E}\right)}{\mathrm{Max}\left({OH}_{E}\right)-\mathrm{Min}\;\left({OH}_{E}\right)}$ , where *OH* is the normalized one-hot encoding data.
Step 4 Split the train and test
    Split the data into sets for training and testing.
Step 5 aiGeneR 3.0 application (train data)
    Customize the model by $aiGeneR3.0=Initialize_{model}\left(\theta \right)$

    Utilizing the training set ${aiGeneR3.0}_{trained}=Train\;\left(aiGeneR3.0,X_{train},Y_{train}\right)$ , Training the aiGeneR 3.0 model.
    Acquire the predictive model. ${aiGeneR3.0}_{predictive}={aiGeneR3.0}_{trained\left(X_{train}\right)}$

    Find out what percentages of the various types are resistant to antibiotics with the Equation $S_{res}=\frac{1}{n}\sum_{i=1}^{n}\left({\hat{y}}_{train,i}=resistant\right)$ , Where ${\hat{y}}_{train,i}=resistant$ is the indicator function, which has values ${\hat{y}}_{train,i}=resistant=\left\{\begin{array}{c} 1,if\;{\hat{y}}_{train,i}is\;resistant\\ 0,if\;{\hat{y}}_{train,i}\;is\;suceptible \end{array}\right.$

Step 6 Multi-drug resistant prediction and identification of resistant strains (test data)
    Determine which strains are resistant in the test data by following the Equation.
    
${\hat{y}}_{test}={aiGeneR3.0}_{predictive}\left(X_{test}\right).$

    Estimate the resistance of strains to multiple drugs as per the following Equation, $S_{multi-drug}=Estimate\_Res\left({\hat{Y}}_{test}\right)$ . Where $Estimate\_Res\left(\;\right)$ determines multi-drug resistant by comparing the predicted resistance probability ${\hat{Y}}_{test}$ against a threshold $T_{res}$ . The rule for identification follows the rule below, $S_{multi-drug,i}=\left\{\begin{array}{l} 1,if{\hat{Y}}_{test,i}\geq T_{res}\;\left(multi-drug\;resistant\right)\\ 0,otherwise\;\left(non-resistant\right) \end{array}\right.$

    Achieve outcomes with ${S\_R}_{classification}=Classify\;\left({\hat{y}}_{test}\right)$ as susceptible-resistant strains.
    
$S_{resistant}=\frac{\sum_{i=1}^{m}1\left({\hat{y}}_{test,i}=resistant\right)}{m}$ , determine the percentage of bacterial strains that are resilient to antibiotics.
Step 7 Assessment of the Model
    Analyze aiGeneR 3.0’s effectiveness.

### 3.7 Architecture and parameter

**FIGURE 5:**
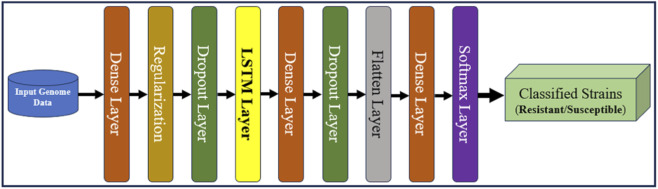
Local architecture of the LSTM deployed in our study.

The primary step in using aiGeneR 3.0 is careful data preprocessing. The genomic sequences of different strains of bacteria are encoded into numerical formats that are suitable for input into neural networks. Enabling further calculation usually involves converting categorical genetic data into a numerical format using methods like one-hot encoding (Dahouda and Joe, 2021). The proposed architecture consists of a total of eight layers, and four types of layers are utilized, out of which three are dense, two are dropout, and one each for regularization, flattening, and softmax make up the model’s architecture. The first, second, and third dense layers contain 64, 64, and 32 neurons, respectively. Comparably, our work employs many values for the dropout and regularization layers. The different regularization values experimented with in our work are 0.01, 0.001, and 0.0001, and the dropout rates are 0.25, 0.5, 0.7, and 0.9. Our proposed local architecture of the LSTM model with other added layers utilizing the random search is shown in Figure 5.

The main objective of this study is to examine and compare the effectiveness of our proposed aiGeneR 3.0 model with different parameters to achieve the best classification accuracy for identifying the resistant strains utilizing the WGS *E. coli* NGS data. Thus, we adopt several changes during the implementation phase to the parameters of aiGeneR 3.0. We discuss some key phases of our experiments in the following sub-sections and the updation in several parameters of our implemented model, among which a few are shown in Table 2.

**TABLE 2:** All the phases of model implementations have different parameter configurations.

Phases	Train-test	Regularization	Dropout	K-fold
1	70:30	0.01	0.25	3
2	**80:20**	**0.001**	**0.5**	**5**
3	90:10	0.0001	0.7	10
4	70:30	0.001	0.5	5
5	80:20	0.01	0.25	5
6	80:20	0.0001	0.5	5
7	80:20	0.001	0.7	10

*Bold showing the best result.

## 4 Experimental setup and implementation

The proposed aiGeneR 3.0 is a complete package of DL and ML models for identifying resistant strains and predicting multidrug resistance in strains. The architecture of aiGeneR 3.0 is simple and less complex than that of previously proposed DL models for resistant strain identification. In our experimental setup, we implemented several versions of aiGeneR 3.0 with different model parameters and finally proposed the architecture that consumes less computational time and produces the most significant results. In this section, we discuss a few of the several implementation versions of aiGeneR 3.0 with different hyperparameters.

### 4.1 Experiment I: high learning rate with smaller training data

During the initial development phase, we are refining the aiGeneR 3.0 model, which employs an LSTM architecture, for our analysis. A softmax layer was incorporated to facilitate classification tasks. A learning rate of 0.01 and a dropout rate of 0.25 were utilized to optimize the training process and mitigate overfitting. To conduct a comprehensive assessment of the model’s performance, we partitioned the dataset into two distinct subsets: the training set and the testing set. The ratio of the train-test split was 70:30. Furthermore, a K-fold cross-validation technique was employed with K = 3 to assess the model’s ability to generalize. Through iterative training and evaluation on various subsets of the dataset, we successfully enhanced the accuracy and dependability of the model in identifying resistant strains in the data.

### 4.2 Experiment II: moderate learning rate with increasing training data

In this implementation phase, we continued our research by iteratively improving the aiGeneR 3.0 model by changing several critical hyperparameters. We adjusted the learning rate to 0.001 to address overfitting and raised the dropout rate to 0.5. These changes should promote more regularization. To keep the assessment process consistent, we partitioned the dataset at an 80:20 train-test split ratio. We also used a K-fold cross-validation method with K = 5 to strengthen our model evaluation and thoroughly examine its generalizability capabilities, which improved the validation procedure. The model’s training dynamics were fine-tuned using these improvements so that it could better use features from the NGS data to identify resistant bacteria.

### 4.3 Experiment III: low learning rate with maximum training data

During this experiment phase, we kept tweaking the hyperparameters of the aiGeneR 3.0 model to make it even better. Now, we are trying to find the sweet spot by gradually adjusting the model weights during training with a learning rate of 0.0001. We raised the dropout rate to 0.7 to improve model regularization and alleviate overfitting worries; this should lead to more diverse and resilient learned representations. To keep things uniform throughout the assessment, we kept the train-test split ratio at 90:10. To further evaluate the model’s efficacy across different data subsets, we also used a more stringent K-fold cross-validation method with K = 10. This broadened the scope of our validation strategy.

We consistently obtained the best performance metrics with an 8:2 train-test ratio throughout all stages of our model, aiGeneR 3.0, as shown in Table 2. This ratio consistently produced the best outcomes, even with changing parameter combinations during the different phases. After training on 80% of the data and testing on the remaining 20%, our model showed exceptional accuracy, precision, sensitivity, and specificity. This strategy ensured that generalization and model complexity were balanced, enabling reliable performance on several splits of the datasets. Furthermore, at each step, regularization strategies, dropout rates, and K-fold cross-validation were methodically investigated to improve the performance of our models. Notably, the 8:2 train-test ratio was a stable base for attaining optimal outcomes across all of our implementations, even though changes to the parameters affected the model’s behavior.

In addition to the above experiments, we also implemented our aiGeneR 3.0 in several other phases, with the model parameters fine-tuned. We also take the different train-test splits to the above experiments and add various other possible dropout rates. However, we observe different model matrices with each of these implementation phases of our aiGeneR 3.0 and consider the best performance, which is described in sections 5 (results) and 6 (discussions).

## 5 Performance evaluation

This section presents a thorough performance evaluation of aiGeneR 3.0 and discusses the various evaluation processes adopted in our study. Our study employs a distinct blend of methodologies: power analysis, empirical analysis, and evaluation of model generalization. Empirical analysis assesses the practical value of the model in real-world situations, while power analysis evaluates its ability to detect meaningful effects. The analysis of model generalization focuses on its ability to acquire knowledge from training data and adjust to various unseen datasets. This comprehensive evaluation technique will unveil the intricate complexities of aiGeneR 3.0, providing insights into its effectiveness and robustness.

### 5.1 Power analysis

Power analysis is a statistical method employed to ascertain the minimal sample size necessary for a study to attain a specified degree of statistical power (Nayak et al., 2024; Jamthikar et al., 2020). Power analysis is essential in the realm of deep learning models as it allows for the estimation of the required sample size to effectively detect significant impacts or disparities in the model’s performance while maintaining a desired level of confidence.

We conducted a power analysis to determine the minimum sample size needed to calculate a population proportion with precision and accuracy. The experiments were conducted utilizing the methodology described in (Jamthikar et al., 2019; Skandha et al., 2020). The formula for sample size calculation, represented by the symbol Sn, is shown in Equation 1:

$$S_{n}=\left[\;{\left(z*\right)}^{2}\times \left(\frac{\tilde{p}\left(1‐\tilde{p}\right)}{{\text{MoE}}^{2}}\right)\;\right]$$ (1)

In this context, MoE represents the margin of error, $\tilde{p}$ denotes the estimated proportion of the feature in the population, and z* refers to the Z-score linked to the relevant confidence level. The MoE2 was calculated by using half of the width of the confidence interval. We selected a ratio of 0.5 and a confidence level of 95% for our experiment. The power analysis is conducted using MedCalc (Medcalc, 2025) and demonstrates that the study has a sample size (809) that exceeds the required amount to achieve the intended degree of statistical power and correct classification. The minimum sample size for the dataset utilized is 271 (68 are susceptible and 203 are resistant), which is smaller than the available data.

### 5.2 Empirical analysis

The confusion matrix, a real-to-anticipated-class matrix with multiple evaluation standards, is the primary target of performance parameters. TP and FP stand for true positives and false positives, respectively, in the confusion matrix. Similarly, TN and FN represent true negatives and false negatives, respectively. There are four types of predictions: TP, which accurately predicts that samples with resistance will be resistant; TN, which accurately predicts that samples without resistance will be susceptible; FP, which inaccurately predicts that susceptible samples will be resistant; and FN, which inaccurately predicts that resistant samples will be susceptible.

Measures such as accuracy (Acc), precision (Pre), specificity (Spe), sensitivity (Sen), F1-score (F1), Matthews Correlation Coefficient (MCC), and area under the curve (AUC) are some of the classification performance measures that were studied in this study. The total number of input samples divided by the number of valid predictions is the “processor, ranging from 1 to 4 (dataset A), while the second dataset consists of one-hot encoding. We call the recall the percentage of positive observations that were projected to be positive compared to the total number of positive observations. F1 is the weighted mean of precision and recall. All the model metrics are calculated based on the following equations (Equations 2–7).

$$\text{Accuracy }\left(\text{Acc}\right)=\frac{\left(T_{P}+T_{N}\right)}{\left(T_{P}+F_{P}+T_{N}+F_{N}\right)}$$ (2)

$$\text{Precision }\left(\text{Pre}\right)=\frac{T_{P}}{T_{P}+F_{P}}$$ (3)

$$\text{Sensitivity }\left(\text{Sen}\right)=\frac{T_{P}}{\left(T_{P}+F_{N}\right)}$$ (4)

$$\text{Specificity }\left(\text{Spe}\right)=\frac{T_{P}}{\left(T_{N}+F_{N}\right)}$$ (5)

$$F1\text{ Score}=2*\;\frac{\text{Precision}*\text{Recall}}{\text{Precision}+\text{Recall}}$$ (6)

$$\text{MCC}=\frac{T_{P}\times T_{N}-F_{P}\times F_{N}}{\sqrt{\left(T_{P}+F_{P}\right)\left(T_{P}+F_{N}\right)\left(T_{N}+F_{P}\right)\left(T_{N}+F_{N}\right)}}$$ (7)

## 6 Results

The Anaconda environment and Jupyter Notebook are used to carry out the model architecture design and parameter configuration. The learning models are implemented in Python (version 3.7) (Python, 2025). Here, we present the findings from the exploratory data analysis, together with a discussion of the results obtained using the suggested methodology. Our study optimized K-Nearest Neighbors (KNN), Decision Tree (DT), Support Vector Machine (SVM), VGG-19, 1-Dimensional Convolutional Neural Network (1-D CNN), and ResNet-50 to ensure resilient performance. Grid search found the best k for KNN, balancing classification accuracy and computing economy. DT used a Gini impurity-based criterion with a maximum depth to avoid overfitting. SVM utilized an RBF kernel with optimized hyperparameters C and γ by cross-validation. Transfer learning adjusted VGG-19 and ResNet-50 architectures were adapted to gene expression data, where both models were trained from scratch with customized input layers and trained using the Adam optimizer. Convolutional, pooling, and dense layers were added to the 1-D CNN architecture with sequential data pattern kernel sizes and activation functions. All models were hyperparameter-tuned and evaluated for optimal performance.

We used a grid search to optimize the learning rate, batch size, hidden layers, and neuron counts hyperparameters for aiGeneR 3.0; we then tested each combination using 5-fold cross-validation to make sure it generalized well and did not overfit. The input data was meticulously preprocessed to remove duplicates, remove samples with too many missing values, and impute missing entries using nearest-neighbor averaging. The magnitude-driven biases in deep learning models were mitigated by scaling numerical features to 0.25–1. To classify resistance, we used a 0.5 threshold to turn projected probabilities into binary calls, and we fine-tuned for unbalanced medicines using ROC to maximize F1-score and minority-class detection. Stable training, repeatable performance, and accurate resistance strain prediction were all achieved by means of this integrated technique.

The proposed aiGeneR 3.0 architecture was constructed using two different machines. The main machine, also known as machine-1, is a workstation running Ubuntu 20.04 that has an Intel Core i7 CPU, 32 GB of RAM, and 1 TB of solid-state drive storage, among other characteristics. The second machine, called Machine-2, is equipped with an Intel Core i5 processor, ranging from 1 to 4 (dataset A), while the second dataset consists of one-hot encoding, utilizing two different datasets. The first dataset consists of the one-hot encoding, ranging from 1 to 4 (dataset A), while the second dataset consists of one-hot encoding, ranging from 0.25 to 1 (dataset B). The comparison of the two systems’ computing time performance using the implemented model is presented in Table 3.

**TABLE 3:** Computational time taken by all the studied models.

Models	Dataset A (µs)		Dataset B (0.25–1) (µs)	
	Machine-1	Machine-2	Machine-1	Machine-2
SVM + RBF	250	420	90	114
DT	241	495	120	151
KNN	306	570	122	142
VGG-19	320	640	120	148
1D-CNN	250	426	95	113
ResNet-50	296	580	123	146
CNN TL	300	620	126	146
CNN Ensemble	380	592	117	141
aiGeneR 3.0	207	370	**87**	**97**

The bold values show the best performance result in our study.

From the above table, we observed notable variations in the computation times of different deployed models when they were assessed during both the training and testing stages, including aiGeneR 3.0. Notably, our suggested aiGeneR 3.0 model leads other studied models in terms of efficiency for the two datasets, consuming just 207 µs for machine-1 and 87 µs for machine-2. Due to its better hardware, machine-1 constantly shows faster computational times than machine-2; yet, aiGeneR 3.0 is the most effective model, with quick processing times that boost output and facilitate quick decision-making. On the other hand, other models like SVM + RBF, DT, KNN, VGG-19, 1D-CNN, ResNet-50, CNN TL, and Ensemble approaches have significantly longer training and testing times on both the datasets studied. All things considered, aiGeneR 3.0’s effectiveness highlights how quickly it can train and assess models, which shows its potential for quick learning capacity. We evaluate our aiGeneR 3.0 with a previously developed TL model. Ren et al. (2022b) and found that it consumes a remarkably less computational time of 31% and 45% in machine-1 for dataset A, and similarly takes 40% and 38% less in machine-2 for dataset B, as seen in Table 3. In addition to this, it can be seen from the table that the one-hot encoding approach adopted in our study (dataset B) shows a remarkably lower computational time compared to dataset A (Ren et al., 2022b) for producing the classification result for all the studied models.

This section quantifies and thoroughly examines the accuracy of the proposed framework, aiGeneR 3.0. Regarding its simple bending model architecture and predictive abilities, aiGeneR 3.0 performs admirably in various tasks, including prediction and classification. The pipeline of aiGeneR 3.0 is the adaptation of the LR and LSTM algorithms. Through an in-depth evaluation of its accuracy, we aim to gain insight into how well aiGeneR 3.0 works when it comes to resistance strain classification with limited computational capabilities and unbalanced data. This section discusses the outcome of our work in the following manner,

A. The ability of our aiGeneR 3.0 model to identify resistant strains by utilizing a single antibiotic.
B. The performance outcome of aiGeneR 3.0 on all four antibiotics taken together to identify the resistant strains.
C. Comparison of all the studied AI models
D. The aiGeneR 3.0 and multi-drug resistance prediction

A: We assess the performance of all our studied models on four different antibiotics. The antibiotics considered for our work are CTX, GEN, CTZ, and CIP. We observed better model metrics while we deployed our proposed model on the CIP dataset, as this dataset is quite balanced compared to other datasets. The detailed model metrics for the CIP dataset of implementations are shown in Table 4 below.

**TABLE 4:** Performance metrics of all the studied models on the CIP dataset.

Model	Acc (%)	Pre (%)	Sen (%)	Spe (%)	F1 (%)	MCC (%)	AUC (%)
SVM + RBF	86	87	86	94	86	81	94
DT	83	84	83	78	83	61	94
KNN	83	83	83	82	83	65	94
ResNet-50	90	90	90	90	90	80	96
VGG-19	82	84	83	91	83	75	90
1D-CNN	86	88	83	88	86	76	91
CNN TL	91	91	89	91	91	82	97
CNN Ensemble	92	92	90	92	91	84	97
aiGeneR 3.0	**93**	**96**	**90**	**95**	**92**	**90**	**99**

The bold values show the best performance result in our study.

**FIGURE 6:**
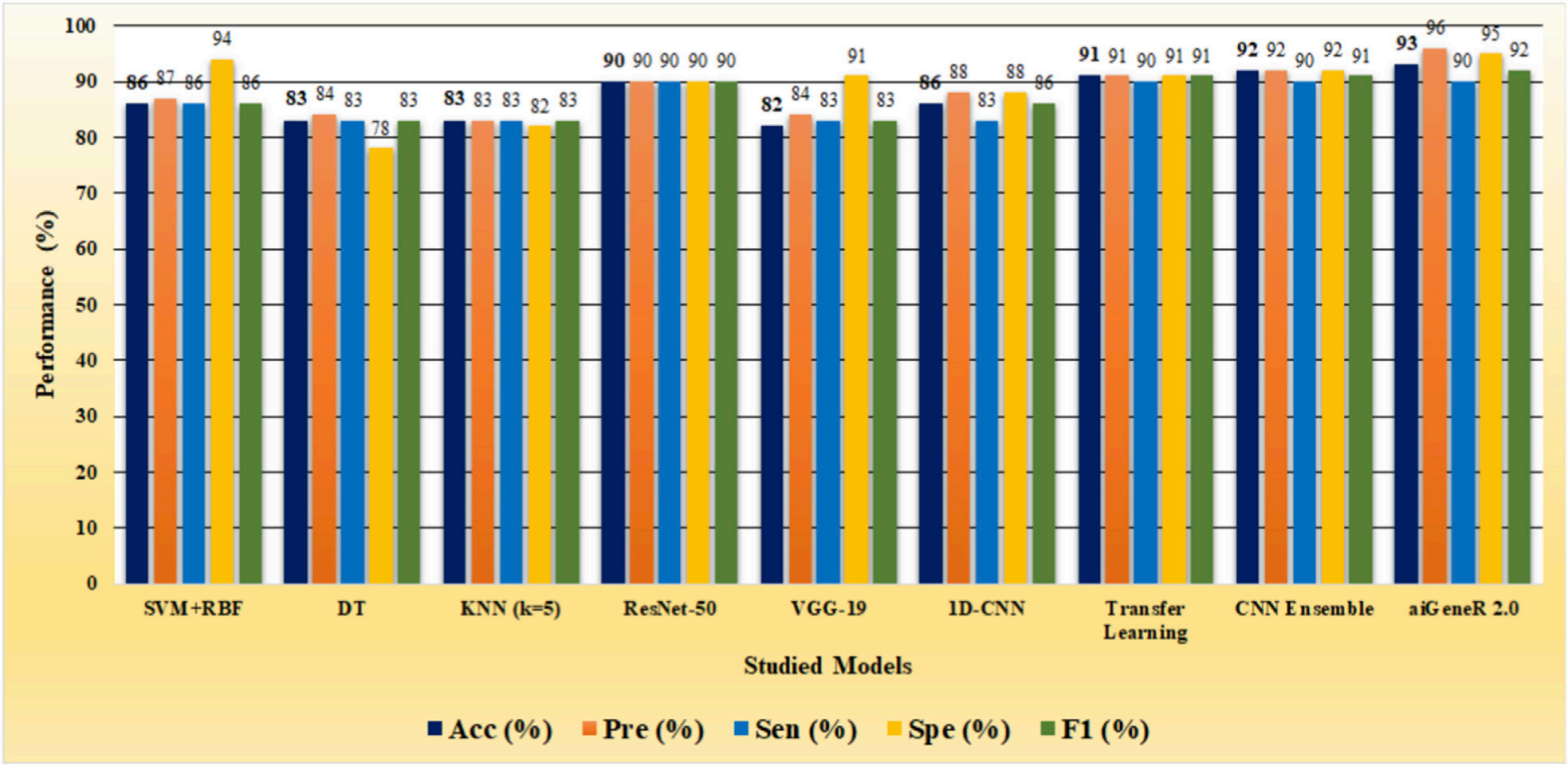
Performance metrics of all the deployed models on the CIP dataset.

The identification of resistant strains by our proposed aiGeneR 3.0, utilizing the CIP dataset, has an accuracy of 93%, which is higher than that of all the studied models. In addition to this, our proposed approach achieves higher sensitivity and specificity of 90% and 95%, respectively, as shown in Figure 6.

We also evaluate our proposed model on the CTX, CTZ, and GEN antibiotics datasets. aiGeneR 3.0 achieves the highest classification accuracy of 82%, 88%, and 80% for CTX, CTZ, and GEN data, respectively. It is observed that the GEN dataset is highly imbalanced and contains a susceptible-to-resistant ratio of 4:1, and notably, our aiGeneR 3.0 reaches the highest classification accuracy of 80% among all the studied models. In addition to this, aiGeneR 3.0 sustains good specificity and sensitivity values for all three antibiotics, which shows its potential to classify the resistant strains with a very minimal false negative rate. The model metrics for CTX, GEN, and CTZ are summarized in Table 5 below. However, among all the studied models, CNN ensemble, CNN TL, and 1D CNN perform better compared to other models in terms of classification accuracy.

**TABLE 5:** Performance metrics of all the studied models on the CTX, CTZ, and GEN datasets.

Model	CTX					CTZ					GEN				
	A (%)	P (%)	Se (%)	Sp (%)	F (%)	A (%)	P (%)	Se (%)	Sp (%)	F (%)	A (%)	P (%)	Se (%)	Sp (%)	F (%)
SVM + RBF	77	77	77	78	77	82	82	81	84	82	74	62	75	20	85
DT	67	67	68	67	67	74	75	78	68	75	66	73	80	34	76
KNN	74	73	75	73	74	79	79	82	73	79	72	79	83	44	81
ResNet-50	75	75	75	75	75	78	79	78	79	78	63	60	88	37	71
VGG-19	72	71	72	71	72	67	70	65	76	62	75	86	77	46	85
1D-CNN	73	73	71	75	74	82	82	87	75	84	79	80	78	50	81
CNN TL	79	81	80	78	80	83	84	88	79	79	79	79	77	47	85
CNN Ensemble	80	81	79	80	80	83	83	89	79	81	80	85	80	60	86
aiGeneR 3.0	**82**	**83**	**93**	**82**	**83**	**88**	**90**	**90**	**84**	**90**	**80**	**91**	**84**	**62**	**87**

The bold values show the best performance result in our study.

**FIGURE 7:**
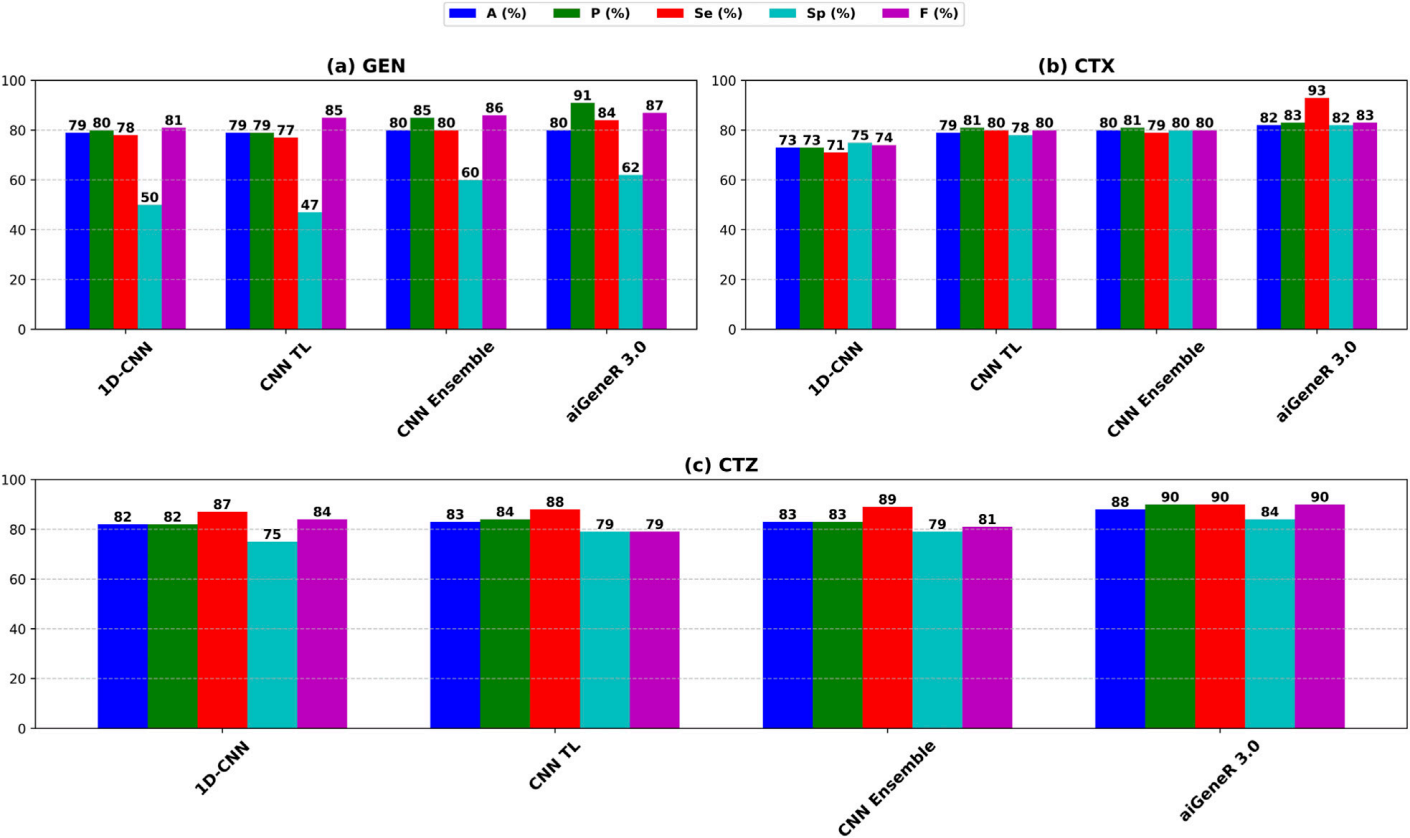
Performance metrics of the top-4 studied models on **(a)** CTX, **(b)** CTZ, and **(c)** GEN datasets.

The performance of aiGeneR 3.0, while we are utilizing the CTX, CTZ, and GEN antibiotics, excels in terms of classification accuracy, sensitivity, and specificity. In addition to this, we obtained a notable sensitivity and specificity while deploying our aiGeneR 3.0 on these three datasets. This result showcases the potential of aiGeneR 3.0 to identify the resistant strains in *E. coli* and can further be tested with other bacterial agents causing antibiotic resistance. The performance of the top-4 models on CTX, CTZ, and GEN datasets based on the accuracy (A), precision (P), sensitivity (Se), specificity (Sp), and F score (F) is visualized in Figure 7.

B: We evaluate the efficacy of our proposed aiGeneR 3.0 to predict the resistance strains by taking all four antibiotics. This pipeline is designed by taking all the strains of the dataset along with all four antibiotics. We refined the dataset by keeping the original susceptible strains and updating the strain resistance to more than two antibiotics as multidrug resistance.

**FIGURE 8:**
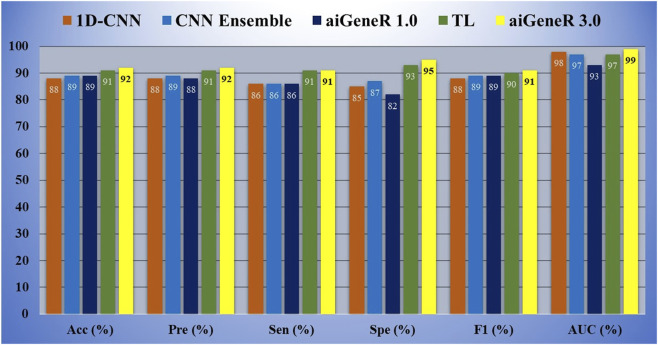
Performance of the top 5 models on all the antibiotics data.

Based on our evaluations with a dataset that included all antibiotics, a learning rate of 0.01, a dropout rate of 0.5, and k-fold cross-validation with k = 5, we found that the aiGeneR 3.0 model performed significantly better than other models. The model metrics for all the studied models are shown in Table 6 below and can be visualized in Figure 8. With an impressive 92% accuracy, 92% precision, 91% sensitivity, and 95% specificity, the model accurately identified resistant strains while reducing false positives and negatives. Additionally, aiGeneR 3.0 demonstrated excellent discriminative ability in differentiating between susceptible and resistant strains with an impressive AUC value of 0.99. These results highlight the efficacy of our model architecture and training methodology, confirming that it is suitable for precise antibiotic resistance prediction and indicating that it may prove to be a helpful tool for improving therapeutic strategies in clinical settings. In addition to this, the classification accuracy of our proposed aiGeneR 3.0 model is 3% higher than that of the previously studied CNN TL model (Ren et al., 2022b). On the highly unbalanced all-antibiotic dataset, the highest Matthews Correlation Coefficient (MCC) obtained by aiGeneR 3.0 is 87%, while the lowest MCC acquired by DT is 28%. Because the CIP dataset is more balanced than the all-antibiotic datasets, the MCC on this dataset is excellent across all models that have been assessed.

**TABLE 6:** Model metrics of all the studied models utilizing the dataset with cases having resistance/susceptibility to all four antibiotics.

Model	Acc (%)	Pre (%)	Sen (%)	Spe (%)	F1 (%)	MCC (%)	AUC	Brier score
SVM + RBF	86	86	86	73	86	56	0.92	0.040
DT	75	75	75	59	75	28	0.72	0.140
KNN	86	86	86	80	86	65	0.89	0.055
ResNet-50	87	87	86	73	86	57	0.90	0.049
VGG-19	82	82	81	76	82	57	0.92	0.047
aiGeneR 1.0	89	88	86	82	89	70	0.93	0.035
1D-CNN	88	88	86	85	88	73	0.98	0.010
CNN Ensemble	89	89	86	87	89	76	0.97	0.015
CNN TL	91	91	91	93	90	84	0.97	0.015
aiGeneR 3.0	**92**	**92**	**91**	**95**	**91**	**87**	**0.99**	**0.005**

The bold values show the best performance result in our study.

C: Comparison of studied models. The studied NGS *E. coli* WGS includes 810 strains and 14,972 SNPs. Our study made use of all 14,972 SNPs with data standardization. With a ratio of 8:2, 648 samples were used for training, and 162 samples were used for testing. The complexity and processing demand of each strategy were evaluated as we explored different models for resistant strain identification using NGS *E. coli* WGS. DT has the potential to overfit as the depth increases, while SVM with RBF kernels is computationally demanding and produces higher classification accuracy compared to DT and KNN, as shown in Table 4. When it comes to prediction, KNN requires more memory and has more computational complexity (Kuang and Zhao, 2009)Thus, we observed a higher computational time for KNN in Table 3. The CNNs like ResNet-50 and VGG-19 deployed in our study have complex architecture and consume more memory and computational cost, as shown in Table 3. The level of complexity in aiGeneR 1.0 is moderate (Nayak et al., 2024). Despite its simplicity, the 1D-CNN still requires a lot of resources. CNN Ensemble adds complexity by combining different models (Zhang et al., 2020) and consumes the highest computational time compared to all the studied AI models, as shown in Table 3. Despite keeping complexity high, CNN TL shortens training time. The proposed aiGeneR 3.0 strikes the perfect balance between processing time and significant classification accuracy, especially due to its streamlined LSTM architecture.

The overall performance of all the models is assessed in terms of classification accuracy, precision, sensitivity, and specificity, as discussed in the performance evaluation section. In addition to this, we consider computational time to be one of the major performance parameters used to evaluate all the studied models. We observe that our proposed model, aiGeneR 3.0, achieves higher performance metrics compared to all other studied models. There is a slight increasing trend in the classification accuracy of aiGeneR 3.0 compared to the previously deployed CNN TL model. Ren et al. (2022b) with a remarkable AUC of 0.99. The most powerful aspect of our aiGeneR 3.0 model is the computational cost; it consumes very little computational power compared to all other studied models. The overall architecture and one-hot encoding techniques adopted by aiGeneR 3.0 make it robust and computationally cost-effective. The multi-drug resistant prediction is one of the significant contributions of aiGeneR 3.0 compared to previous works (Ren et al., 2022a), and it will be discussed in the next section. Additionally, the computational time taken by our aiGeneR 3.0 is much lower compared to all other studied models. The average learning time taken by aiGeneR 3.0 is 86µs less taken from all the studied models together and 83µs less compared to the previously studied TL model.

### 6.1 The aiGeneR 3.0 and multi-drug resistant prediction

Our proposed aiGeneR 3.0 model’s experimental results show potential in predicting multi-drug resistant (MDR) in *E. coli* strains. We considered the strain that resists more than two antibiotics to be in the multidrug-resistant category. We used the prediction model of logistic regression (Vermeiren et al., 2007) to estimate the percentage of bacteria resistant to four frequently given antibiotics: CIP, CTX, CTZ, and GEN. The percentage of resistance to each antibiotic is determined by counting the number of antibiotics that the strain is resistant to; the range is 0.25 for resistance to one antibiotic and 1 for resistance to all four antibiotics. The performance of our deployed resistant prediction model achieves a prediction accuracy of 98%, and the other model metrics for all the studied models are shown in Table 7.

**TABLE 7:** Predictive model metrics for MDR (all studied models).

Model	MSE (Train)	R^2^	RMSE	MSE (new data)
SVM + RBF	0.00349	0.920	0.059	0.00380
DT	0.00680	0.800	0.082	0.00700
KNN	0.00300	0.930	0.055	0.00320
ResNet-50	0.00288	0.940	0.053	0.00300
VGG-19	0.00379	0.910	0.062	0.00400
aiGeneR 1.0	0.00141	0.960	0.037	0.00160
1D-CNN	0.00090	0.980	0.030	0.00100
CNN Ensemble	0.00081	0.985	0.028	0.00090
CNN TL	0.00070	0.990	0.027	0.00080
aiGeneR 3.0	**0.00054**	**0.994**	**0.023**	**0.00051**

The bold values show the best performance result in our study.

The experimental result for MDR prediction witnessed a 98% accuracy rate; our model demonstrated exceptional predictive performance and resilience in detecting variations in MDR. The model’s lowest mean squared error (MSE) during training (0.00054) was found during the model performance evaluation, demonstrating how closely the predicted resistance percentages matched the actual values. Moreover, the high R-squared value of 0.9940 indicates that our model may explain a considerable amount of variability in the resistance percentages across strains. The model’s accuracy for predicting levels of resistance is further demonstrated by the root mean squared error (RMSE) of 0.02327.

Our model’s active predictive capacity was tested using fresh data, and its MSE of 0.00051 confirmed its generalizability and dependability in practical settings. Together, these findings highlight the precision and effectiveness of our suggested aiGeneR 3.0 model in identifying multi-drug resistance in *E. coli* strains, providing crucial data for directing antibiotic treatment procedures and battling antibiotic resistance. As we obtained the best version of our proposed aiGeneR 3.0 with a moderate learning rate, with an increase in training data (80%), we intended to keep this for multi-drug identification. In addition to this, the model parameters like learning rate, CV, train-test split, and dropout rate of aiGeneR 3.0 are 0.001, 5, 8:2, and 0.5, respectively.

## 7 Validation

Accurate evaluation of model performance is a crucial component in building reliable and efficient prediction models. The ability to assess how well a model performs is an important indicator of its suitability for solving practical issues in many fields, including ML and scientific inquiry (Bellazzi and Zupan, 2008). In this section, we take a close look at our proposed models and evaluate them thoroughly, taking into account many criteria so that users can understand their strengths and weaknesses. We examine numerous critical aspects to evaluate the model’s performance in different contexts. Each section delves into a different facet of the model’s performance and thoroughly analyzes its efficacy.

### 7.1 Effect of Training size

The comparison of classification accuracy on test data and all conceivable train-test splits on the used dataset is shown in Table 8. According to the PE (section 4), the objective is to monitor the effect of data size on the model’s performance. As the proportion of training data increases, the accuracy of the aiGeneR 3.0 classification model rapidly increases. It is observed that the model achieves its highest accuracy of 92% when the train-to-test split ratio is 80:20, as shown in Table 8.

**TABLE 8:** Minimum unseen cases and samples are required for the generalization of individual models.

Model	# Unseen samples	# Cases
SVM + RBF	80	648
DT	80	648
KNN	80	648
ResNet-50	80	648
VGG-19	80	648
aiGeneR 1.0	80	648
1D-CNN	80	648
CNN Ensemble	80	648
CNN TL	80	648
aiGeneR 3.0	**70**	**567**

The bold values show the best performance result in our study.

During our experiments, we observed that the studied AI models require more training data for generalization compared to our proposed aiGeneR 3.0 model. If we set the performance threshold as classification accuracy, then our aiGeneR 3.0 takes only 70% (567 unseen cases) of the data to achieve this trademark. Similarly, all of the implemented ML and DL models take 80% of the unseen data to obtain the generalization standard. The generalization of our aiGeneR 3.0 requires 10% less unseen data to obtain the best classification accuracy, proving that our model can be generalized by utilizing fewer strains than all other studied models.

### 7.2 Confusion matrix

**FIGURE 9:**
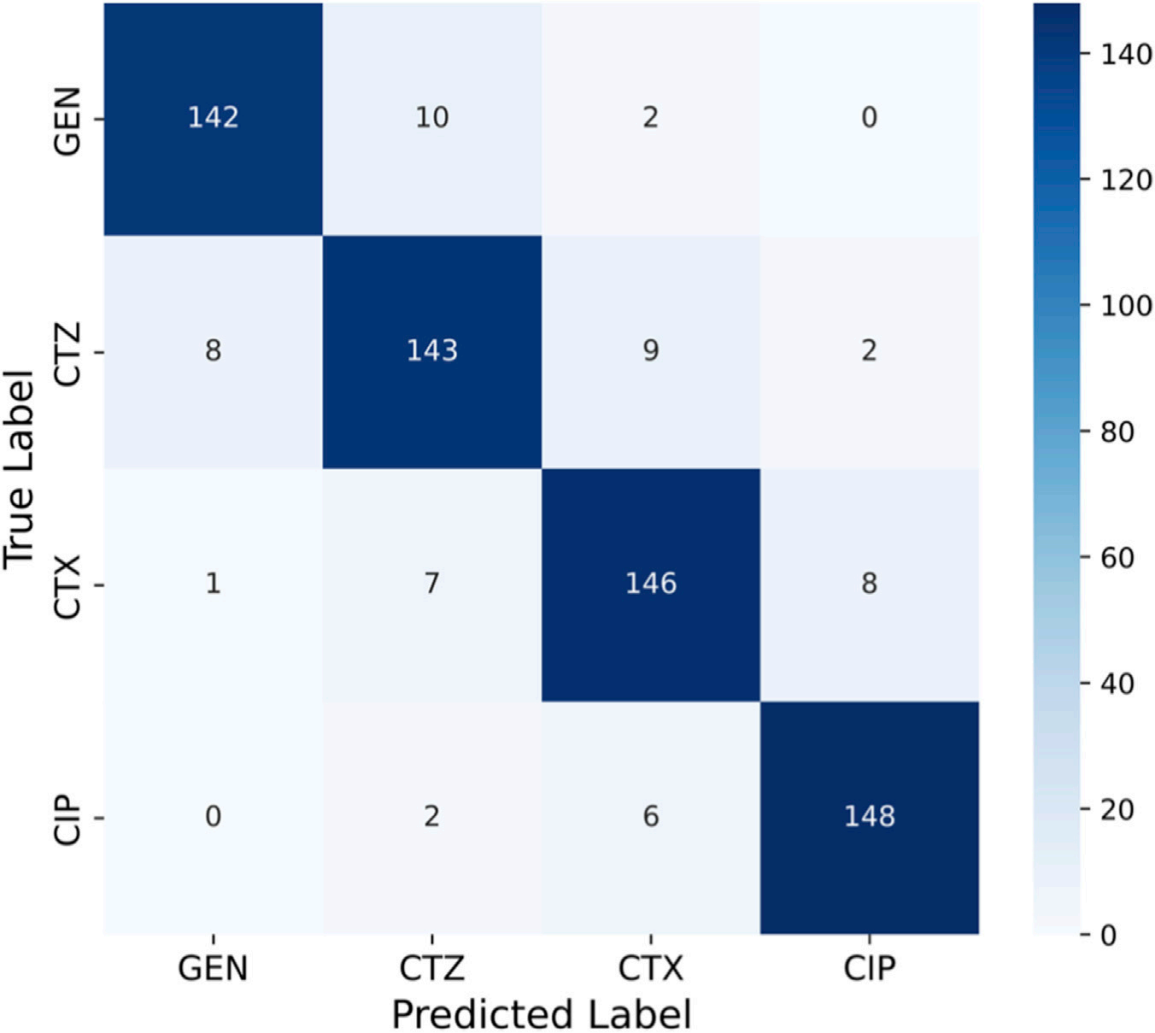
Confusion matrix of aiGeneR 3.0 on all four antibiotics.

The matrix shown in Figure 9 has significant diagonal dominance, indicating that the model predicted the proper class with few misclassifications. Most GEN-resistant strains (142 of 154) were correctly classified, with only a few CTZ and CTX misassignments. The model also predicted CIP, a smaller class, with great accuracy (148 out of 156 properly classified), demonstrating its class imbalance resilience. The confusion matrix yielded class-wise measurements. Each class has good precision, sensitivity (recall), and specificity, indicating that the model minimized false positives and negatives. Minority class CIP had good sensitivity and specificity, showing that the model did not underperform on underrepresented categories, a major antimicrobial resistance prediction difficulty.

The model for GEN has lower specificity (76%) than sensitivity (93%), suggesting reliable identification of susceptible strains but a little probability of under-detection of resistant isolates. CTZ had 91% sensitivity and 85% specificity, recognizing resistant bacteria with minimal false-positive rates. CTX and CIP had a stable finding, with sensitivity and specificity exceeding 92%, indicating robust class classification. CIP’s sensitivity (93%) and specificity (94%) were the best, detecting resistant bacteria and identifying vulnerable ones. These results show that aiGeneR 3.0 consistently supports better specificity while maintaining excellent sensitivity, ensuring reliable detection of minority resistant strains without inflating misleading resistance predictions. The confusion matrix confirms that aiGeneR 3.0 demonstrated balanced predictive performance among antibiotics, with ∼92% accuracy, 92% precision, 91% sensitivity, 95% specificity, 91% F1-score, and 87% MCC.

### 7.3 Receiver operating curves

The Receiver Operating Characteristic (ROC) curve is an essential metric for evaluating the effectiveness of a classification model. In this study, we conduct a performance analysis of our suggested aiGeneR 3.0 model in comparison to other studied models, with a significance level of p = 0.001. K-5 cross-validation is employed to determine the variation in the accuracy of each model as the quantity of training data changes.

**FIGURE 10:**
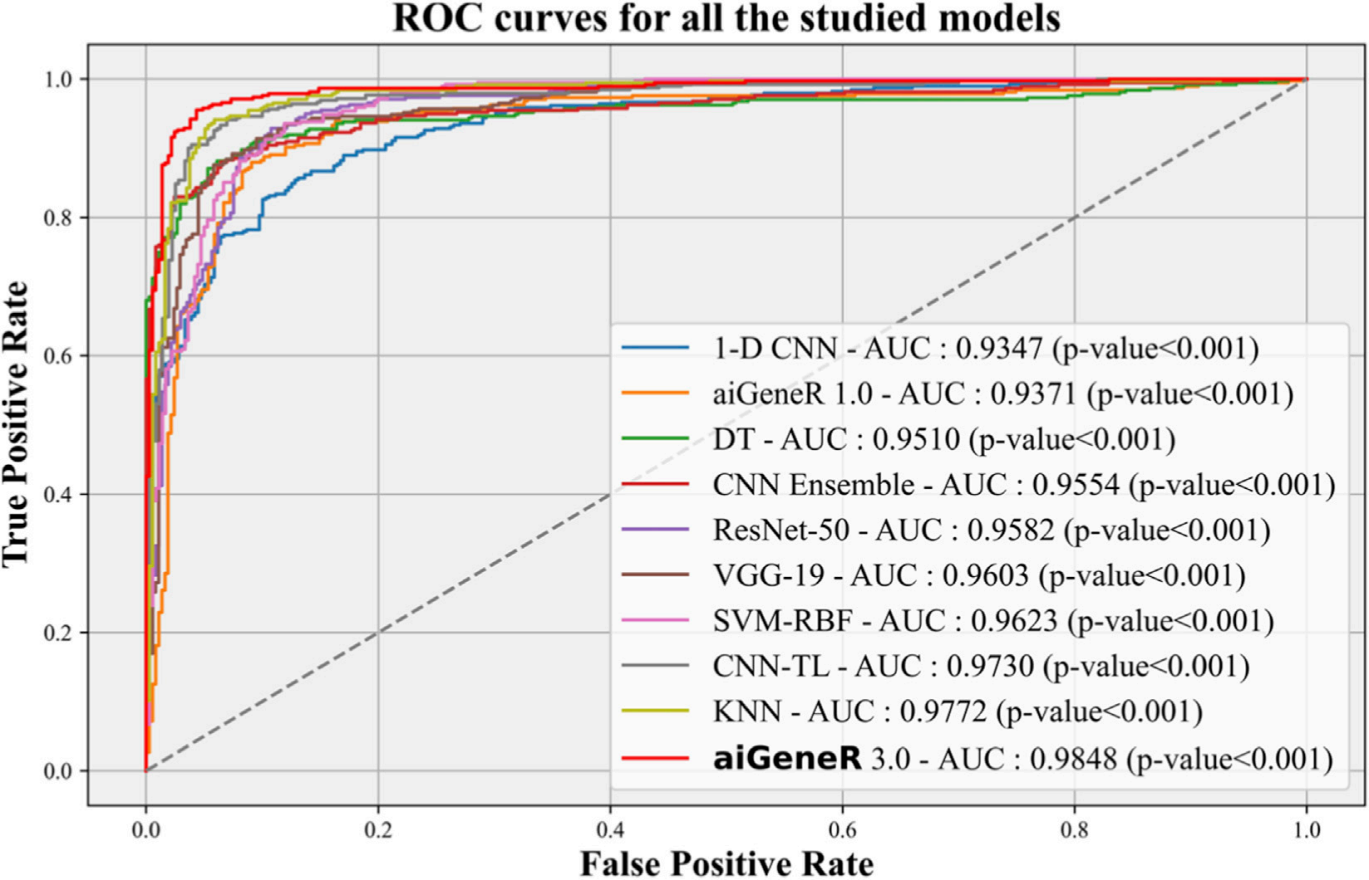
ROC-AUC of all the studied models with p-value <0.001.

The ROC performance of all the studied models is shown in Figure 10. The proposed model, aiGeneR 3.0, has achieved a significant milestone by achieving a strong Area Under the Curve (AUC) value of 98.48%. Nevertheless, when compared to other classification models, the ROC value of the 1-D CNN is the lowest (93.47%). Compared to the previously developed CNN TL model and despite the hurdles posed by the imbalance and small dataset, our proposed aiGeneR 3.0 achieves the highest AUC value in the identification of the resistant strains.

### 7.4 Model generalization

In the validation phase of our aiGeneR 3.0 model, we utilize an openly available and highly imbalanced dataset (Moradigaravand et al., 2018). The detailed characteristics of the dataset are summarized in Table 9. There is a high imbalance in the susceptible-to-resistant ratio in all four antibiotics taken for validation of our aiGeneR 3.0 model. It can be seen from the table that the ratio is very high in the case of CTZ and GEN (≈1:7) there is a slight increase in the ratio for CTX and CIP (≈1:4). We perform the model validation in two different phases first, we have considered four different datasets based on four individual antibiotics and secondly, prepare the dataset by combining all the four antibiotics into one dataset.

**TABLE 9:** Characteristics of the validation dataset.

Antibiotics	GEN	CTZ	CTX	CIP
# Susceptible	1,651	1,670	1,476	1,508
# Resistance	284	265	459	427
Total	1,935	1,935	1,935	1,935

We tested the efficacy of our proposed model on the four individual antibiotics considered for our experiments in the publicly available data, and aiGeneR 3.0 holds the consistency and remains the best performer in terms of classification accuracy, specificity, and sensitivity. In Table 10, we summarize the performance of aiGeneR 3.0 on individual datasets.

**TABLE 10:** Validation model metrics of aiGeneR 3.0.

Dataset	Acc (%)	Pre (%)	Sen (%)	Spe (%)	F1 (%)	MCC
CIP	89	94	92	70	93	0.60
CTX	93	98	94	88	96	0.74
CTZ	90	97	91	79	94	0.60
GEN	89	96	91	77	94	0.59

It is observed from the above table that, during validation of aiGeneR 3.0 with individual antibiotics data, we obtained a higher classification accuracy in the case of CTX, and this is due to the higher strain ratio compared to the other three antibiotics datasets. aiGeneR 3.0 achieves the second-highest classification accuracy in the case of CTZ (90%), followed by CIP and GEN (89%).

**FIGURE 11:**
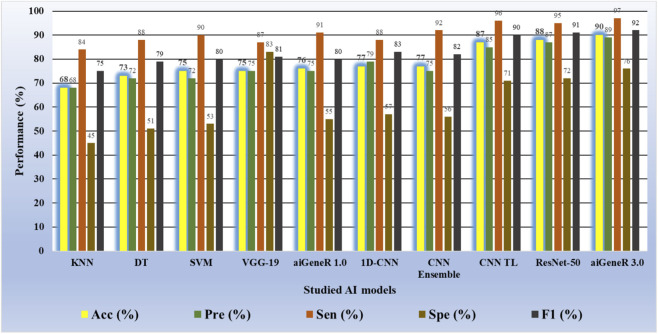
Model metrics of all the studied models during the model validation phase.

Similarly, while we tested aiGeneR 3.0 along with other studied models on the dataset that combines all four antibiotics, we observed that aiGeneR 3.0 achieves the highest classification accuracy (90%), as shown in Table 11. The sensitivity and specificity of aiGeneR 3.0 are 97% and 76%, respectively, which is the highest among all the studied models, and this is due to the one-hot encoding we adopt in our study. In addition to this, the SVM, aiGeneR 1.0, CNN TL, CNN ensemble, and ResNet-50 achieve a remarkable sensitivity of more than 90%, whereas CNN TL and ResNet-50 achieve a specificity just higher than 70%. The studied model metrics of the validation phase are shown in Figure 11. However, in the validation phase, we observed that the ResNet-50, CNN ensembled, and TL performed better than aiGeneR 1.0. This is due to the effectiveness and automated feature extraction techniques with these models compared to our previously developed aiGeneR 1.0, which is based on traditional feature selection techniques. This validation outcome may provide insight into the use of automated and effective feature selection techniques, especially DL, for future resistant strain identification.

**TABLE 11:** Model metrics of all the studied models on the validation dataset (all four antibiotics).

Models	Acc (%)	Pre (%)	Sen (%)	Spe (%)	F1 (%)
KNN	68	68	84	45	75
DT	73	72	88	51	79
SVM	75	72	90	53	80
VGG-19	75	75	87	83	81
aiGeneR 1.0	76	75	91	55	80
1D-CNN	77	79	88	57	83
CNN Ensemble	77	75	92	56	82
CNN TL	87	85	96	71	90
ResNet-50	88	87	95	72	91
aiGeneR 3.0	**90**	**89**	**97**	**76**	**92**

The bold values show the best performance result in our study.

**FIGURE 12:**
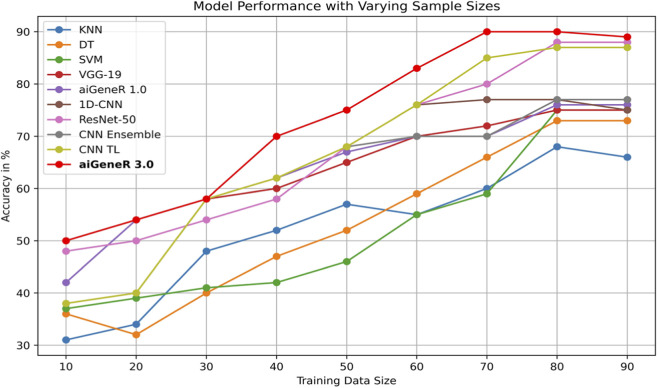
Performance of all the studied models with different train-test splits.

The performance of the designed model on every conceivable train-test split and the comparison of classification accuracy on test data were also explored in this study. The learning model is impacted by the amount of training data, which also helps the model generalize effectively to new data. Using a dataset with various train-test splits, we assess our suggested model, aiGeneR 3.0, and the four other classifiers employed in this investigation. It has been noted that while other models require a more significant number of cases for generalization, aiGeneR 3.0 requires a small number of cases. This section thoroughly explains how data size affects our suggested model. Figure 12 displays the comparison of classification accuracy on test data as well as all conceivable train-test splits on the utilized dataset.

It can be observed from the figure that the ML models require more training samples to obtain generalization in classification accuracy than the DL models. While we compare the top three ML (SVM + DT + KNN) with the top three DL (CNN TL + aiGeneR 1.0 + aiGeneR 3.0) models, there is a significant difference in the train-test split for learning models to achieve their best results. Compared to the top three DL models, the top three ML models take 65.9% more data to be generalized. The other DL models studied in this work take a range of 55%–75% unseen cases to obtain their generalization. In addition to this, our proposed aiGeneR 3.0 requires 70% (567 cases) of data to generalize and obtain a stable classification accuracy. This performance outcome of aiGeneR 3.0 showcases the model’s generalization ability with a very small number of unseen data, which leads to its chances of better performance with real-time data.

## 8 Discussion

The results show that the aiGeneR 3.0 model effectively detects resistance strains without using any known resistance strains during model training. However, there are some limitations to be aware of due to variations in dataset sizes and methods. We implemented our suggested aiGeneR 3.0 model using a basic model architecture and then applied it to a publicly available, imbalanced, and noisy dataset. When given balanced antibiotic data, learning models perform much better in terms of accuracy, and we fine-tuned aiGeneR 3.0 to consistently classify each drug. In comparison to other conventional ML models used in our study, aiGeneR 3.0’s computational time is much lower.

We observed that, because typical one-hot encoding introduces a relative scale with numbers like 1, 2, 3, and 4, higher numerical values may inadvertently dominate or introduce bias during the learning process in certain deep learning models. Using bigger numerical representations may result in learning disparities in certain deep learning models, especially those that are sensitive to input magnitudes (models that ineffectively normalize weights), even if one-hot encoding is categorical and theoretically scale-invariant. By limiting the range to 0.25–1, biases resulting from magnitude are less likely to occur, and a more consistent expression is assured. We found that scaling to a smaller range improved training convergence and made the gradient updates of our models more reliable.

The deployed aiGeneR 3.0 model has a straightforward design that can deliver good classification accuracy. Among the most advanced ML and DL models we tested, aiGeneR 3.0 yielded the best classification accuracy. The following is a list of some of the major study findings we came across while doing this work: a simple and effective model architecture can achieve better classification accuracy, minimal computational cost, antimicrobial resistance (AMR) analysis, and antibiotic resistance strain identification.

• The proposed aiGeneR 3.0 has a simple deep network architecture and has the potential of a good learning model by providing relatively higher classification accuracy to identify the resistant strains.
• The aiGeneR 3.0 requires less computational time compared to all the studied models in this work.
• The multi-drug prediction ability with significant minute errors is a major contribution of our proposed aiGeneR 3.0 model.
• The aiGeneR 3.0 can effectively identify the resistant strains with a classification accuracy of 92% which is the highest among all the studied models.
• Model generalization of aiGeneR 3.0 persists in its classification potential and proves the ability of our proposed model to handle imbalanced and unseen data.

### 8.1 Claim

Our cutting-edge study reveals that aiGeneR 3.0 is an excellent resource for identifying strains of antibiotic resistance; it can handle imbalanced and constrained datasets with ease. Through the utilization of sophisticated DL algorithms, aiGeneR 3.0 achieves better classification accuracy, as shown in Table 12.

**TABLE 12:** Benchmarking parameters of the studied state-of-the-art ML and DL techniques for AMR analysis.

Authors	Objective	Dataset	Techniques	Performance evaluation	Limitations
Pesesky et al. (2016)	Predict AR patterns in Bacilli	Genome Sequence	ML and Rule-based approaches	Acc (Resfams = 94.9%, Resfinder = 85.9%, CARD = 57.7%	The accuracy and generalizability of estimations are affected by constraints such as small sample sizes
Moradigaravand et al. (2018)	Identify AR in *E. coli* bacteria	*E. coli* strains	LR, RF, Gradient Boosting	Acc = 97%, Precision = 93%, Recall = 83%	A high false negative rate caused by undetected SNPs in specific locations and the accessory genome
Kavvas et al. (2018)	Detecting microbial tuberculosis ARG.	Sequence	Pan-genome Analysis, SVM, LR	AUC = 0.80	The challenges include reference bias, data selection bias, and the necessity for experimental validation
Liu et al. (2020)	Predicting the AR in A. pleuropneumonia	Genome sequence	SVM, SCM	Tet (Acc = 97%), Amp(Acc = 100%), Sul (Acc = 100%)	Problems with the size of the dataset, biases, and the generalizability of the model
Green et al. (2022)	Prediction of M.tuberculosis ARGs	WGS	CNN	AUC for MD-CNN = 91.2% AUC for SD-CNN = 93.8%	A higher computational cost is required to validate the results
Li et al. (2022)	Identification of new antimicrobial peptides	Sequence	AMPlify, Bi-LSTM, RNN, CNN	Acc = 93.71%, F1-Score = 93.66%, AUROC = 98.37%	Inadequate training data is the reason for the difficulties in training AMP models
Florensa et al. (2022)	Identifying ARGs in NGS data	NGS data	ML, NGS	-	An issue with the study’s findings is that it lacks any data for the ResFinder tool’s performance parameters, such as sensitivity, specificity, and precision
Ren et al. (2022b)	Predicting AMR.	WGS (*E. coli*)	CNN	CIP (Acc = 91%), CTX (Acc = 78%), GEN (Acc = 78%)	Issues with computing resources, interpretability, external validation, and dataset size
Grey et al. (2023)	UTI current condition review	-	Culture, AI	Acc = 93.22%–98.80%	This study fails to determine treatment accuracy and ARG identification
Almaghrabi et al. (2024)	Analyze resistance genes in *P. aeruginosa*	WGS	Web-based tools	MDR = 77.1%	This study lacks the ability to draw clear epidemiological connections between environmental and clinical isolates
Jin et al. (2024)	AMR prediction in *E. coli*	WGS	RF, SVM, LR, CNN	F1-Sc:82%, MCC:48%, AUC:77%	The learning rate of the model is very low
Gao et al. (2024)	AMR in A. baumannii	Sequence	ML model	Acc = 96%	The analysis limits may affect the model’s generalizability, misbalancing, etc.
aiGeneR 3.0 [Proposed]	Predict MDR and antimicrobial-resistant strains	WGS (*E. coli*)	ML/DL	Acc = 93%, Sen = 90%, Spe = 95%, ROC = 99%	Data augmentation and advanced computational techniques, such as a transformer, may play a crucial role in increasing classification accuracy

By minimizing variance and keeping feature scaling consistent, this method stabilizes the training process, which in turn produces smoother gradients and avoids problems like bursting gradients (dos Santos and Papa, 2022). We found that our studied DL models performed much better when we used a one-hot encoding range of 0.25-1 rather than 1–4. With a 2% increase in specificity and a 1% improvement in precision, our empirical data demonstrated better accuracy and generalizability on both the validation and test sets. In addition, our model was able to generalize well to a different dataset (Moradigaravand et al., 2018), which further proves how effective and resilient this proposed normalized feature range is for the learning of the deployed DL models.

The validation confirms the edge of aiGeneR 3.0, demonstrating its capacity to surpass rivals with small input data. Its processing cost is minimized, and its simple design gives it the ability to run on typical personal computers and laptops, ensuring better classification accuracy. Furthermore, a comprehensive power analysis reveals aiGeneR 3.0’s capacity to surpass the desired number of training cases, underscoring its potential for further refinement and expansion. Additionally, the significant AUC value of 98.48% shows the potential of our aiGeneR 3.0 toward its adaptability and learning capacity with imbalanced datasets. Overall, our research shows that aiGeneR 3.0 is an innovative breakthrough that will change how we diagnose diseases and, more generally, not just when identifying strains of antibiotic resistance.

### 8.2 Special notes

We designed the cutting-edge aiGeneR 3.0 model, a DL-based AMR analysis tool, to use double-mutated gene data to predict multi-drug resistance and detect antibiotic resistance strains without prior knowledge of known ARGs. The powerful DL model, LSTM, and LR combination in aiGeneR 3.0 advances AMR analysis, particularly multi-drug prediction. The primary notable accomplishments of our aiGeneR 3.0 framework are as follows:

• We proposed aiGeneR 3.0, an AI model with a simple and robust architecture that can handle imbalances and small genomics data.
• The aiGeneR 3.0 can predict the resistant strain from a double-mutated gene sequence with higher classification accuracy compared to previous studies.
• aiGeneR 3.0 offers an ultimate ability to predict the multi-drug resistance in strains with 98% prediction accuracy.
• The generalization and scientific validation of aiGeneR 3.0 prove its potential to handle small and imbalanced (curse of dimensionality) gene data.
• The benchmarking of aiGeneR 3.0 with other state-of-the-art- AI models enhance its adaptability for real-time implementation.

This proposed aiGeneR 3.0 model has the ability to identify *E. coli* bacteria that are resistant to antibiotics, which could be useful for antimicrobial stewardship initiatives. The approach has the potential to lower the usage of inefficient medicines and limit the use of broad-spectrum antibiotics by offering early insights about resistance profiles, which could support antibiotic selection that is tailored to individual patients. Subject to medical validation, the fast prediction capacity suggests real-time clinical decision support. Hospital monitoring systems can benefit from aiGeneR 3.0, which could have applications beyond individual patient care and bolster initiatives to combat new resistance tendencies. However, before these applications are widely used in ordinary practice, more multicenter clinical trials and prospective validation must be conducted.

### 8.3 Limitations

This highly motivated study focuses on identifying resistant strains utilizing imbalances and small datasets. Data augmentation can be used to further this study and potentially improve model performance. However, because it is medically incorrect, experts do not advise using this approach (the augmentation of medical data). Better model metrics might be obtained if the model were trained using synthetic data. Further study could address a few biases in our model, such as (i) smaller studies are found related to our work, (ii) SNP filtering threshold applied during preprocessing, which may have influenced the set of variants included for model training (iii) the use of data augmentation, (iv) comparisons with other ML and recently trending DL models like deep network with an attention mechanism. (v) a summary of the benchmarking studies and (vi) no remarks regarding the clinical validation (Paul et al., 2022; Eskofier et al., 2016; Hu et al., 2021).

In addition to the above, AiGeneR 3.0 has a few limitations despite its better predictive accuracy. First, despite balancing and preprocessing, the datasets had class imbalances that potentially bias predictions toward the majority class. The model may learn dataset-specific artifacts instead of generalizable biological patterns when training on short or noisy datasets, increasing the risk of overfitting.

### 8.4 Extension

This work focuses on applying DL and AI models to resistant strain identification and classification. The proposed aiGeneR 3.0 is now considered a benchmark in the field of AMR analysis due to its great improvement in detecting resistant strains. The aiGeneR 3.0 model performs highly compared to earlier research (Ren Y. et al., 2022) on resistant strain identification. Furthermore, cross-validation and unseen implementations show the system’s endurance, domain flexibility, and capacity to function well in domains other than the one on which it was trained. In extension, the application of other DL models, especially transformer architecture with attention mechanism, and Dl model hyper-parameter optimization may be adopted to validate their efficacy in identifying multi-drug resistance in double-mutated genome sequences.

Despite aiGeneR 3.0’s capabilities, small or imbalanced datasets risk overfitting, when the model learns training data-specific patterns or noise rather than generalizable correlations. Even high accuracy on the training set may not guarantee accurate predictions on unseen data. Imbalanced class distributions bias the model toward majority classes, making unusual antibiotic-resistant organisms harder to detect. Additional variability can worsen model performance in real-world deployment. Different laboratory techniques, sample preparation methods, sequencing platforms, and batch effects introduce systematic variances that may not be in the training data. Sequencing errors or missing data can skew input features, and variable sample distributions across populations may produce patterns the model has not learnt, raising misclassification risk. Parameters and techniques like Cross-validation, data augmentation, and regularization (dropout, weight decay, and early stopping) need to be tested in a wider range. Finally, ongoing retraining with new datasets adapts the model to changing data distributions, ensuring robustness and reliability in clinical or laboratory contexts. Future research should use explainable AI methods like feature attribution or pathway-level analysis to identify resistance-predicting genes or biological markers. For clinical implementation, the model needs to be verified across larger, multi-center cohorts to account for sequencing procedures, sample handling, and patient demographics. To maintain accuracy and dependability in clinical operations, rigorous benchmarking, seamless software interfaces, real-time processing, and constant retraining with new resistance data are needed.

## 9 Benchmarking

At its core, our study centered on identifying resistant strains using a DL model that combined advanced techniques with a simple design. Along with this, we also want to make sure that our pipeline does not lose consistency when applied to small or imbalanced datasets. Research shows that few studies have used DL models to identify resistance strains in double-mutated WGS. This set of AI models was constructed by merging several deep networks. Hence, it is essential to evaluate our method for previous AI models. In light of this, we choose to tackle the benchmarking efforts head-on by comparing our proposed models to earlier DL/ML models used in AMR for resistant strain classification and other disease investigations.

Our suggested aiGeneR 3.0 has a higher classification accuracy of 93% and can manage imbalanced data because of its streamlined model architecture. Furthermore, the computing time required for both the learning phase and the prediction of resistant strains in aiGeneR 3.0 is significantly lower compared to previously examined DL models. Furthermore, the validation of aiGeneR 3.0 establishes it as a strong and versatile model for classifying antibiotic-resistant strains and predicting multidrug resistance.

## 10 Conclusion

In this work, we used double-mutated *E. coli* NGS WGS data to show how effective aiGeneR 3.0 is in identifying bacteria that are resistant to antibiotics. Additionally, it presents the multi-drug-resistance patterns identified in the resistant strains. aiGeneR 3.0 is a hybrid computational method that employs advanced LSTM architecture and NGS data to discern resistant and susceptible strains within small and highly unbalanced datasets. Primarily, our aiGeneR 3.0 model enhances the accuracy of classification and prediction compared to earlier investigated models. The remarkable performance of our proposed pipeline is evidenced by aiGeneR 3.0, achieving a 92% accuracy, with a sensitivity of 91% and a specificity of 95% in finding resistant strains. aiGeneR 3.0 attains a 98% prediction accuracy in multi-drug identification, accompanied by a minimal MSE of 0.00054 and an RMSE of 0.02327.

Our study emphasizes the promise of predictive modelling utilizing NGS data and DL techniques to tackle the escalating problem of antibiotic resistance, perhaps leading to the creation of novel therapies. The ability of aiGeneR 3.0 to consistently and extensively generate models indicates its prospective usefulness in AMR research moving forward. Antibiotic resistance is emerging as a critical concern in the field of infectious diseases. Our research enhances our comprehension of the issue, enables us to predict its future trajectories, and eventually aids in addressing it. Due to the numerous constraints in identifying resistance patterns across different strains resulting from the limited number of strains, we want to employ deep learning models on whole genome sequencing with various augmentation techniques in our future research to find resistant strains.

## Data Availability

The original contributions presented in the study are included in the article/supplementary material, further inquiries can be directed to the corresponding authors.
